# Intra-limb modulations of posterior root-muscle reflexes evoked from the lower-limb muscles during isometric voluntary contractions

**DOI:** 10.1007/s00221-021-06187-5

**Published:** 2021-08-06

**Authors:** Akira Saito, Kento Nakagawa, Yohei Masugi, Kimitaka Nakazawa

**Affiliations:** 1 Center for Health and Science, Kyushu Sangyo University, Matsukadai, Higashi-ku, Fukuoka, Japan; 2 Graduate School of Arts and Sciences, The University of Tokyo, Komaba, Meguro, Tokyo Japan; 3 Japan Society for the Promotion of Science, Kojimachi, Chiyoda, Tokyo Japan; 4 Faculty of Sport Sciences, Waseda University, Mikajima, Tokorozawa, Saitama Japan; 5 Institute of Sports Medicine and Science, Tokyo International University, Matoba, Kawagoe, Saitama Japan

**Keywords:** Corticospinal excitability, Spinal reflex excitability, Electromyography, Transcranial magnetic stimulation, Spinal cord stimulation

## Abstract

Although voluntary muscle contraction modulates spinal reflex excitability of contracted muscles and other muscles located at other segments within a limb (i.e., intra-limb modulation), to what extent corticospinal pathways are involved in intra-limb modulation of spinal reflex circuits remains unknown. The purpose of the present study was to identify differences in the involvement of corticospinal pathways in intra-limb modulation of spinal reflex circuits among lower-limb muscles during voluntary contractions. Ten young males performed isometric plantar-flexion, dorsi-flexion, knee extension, and knee flexion at 10% of each maximal torque. Electromyographic activity was recorded from soleus, tibialis anterior, vastus lateralis, and biceps femoris muscles. Motor evoked potentials and posterior root-muscle reflexes during rest and isometric contractions were elicited from the lower-limb muscles using transcranial magnetic stimulation and transcutaneous spinal cord stimulation, respectively. Motor evoked potential and posterior root-muscle reflex amplitudes of soleus during knee extension were significantly increased compared to rest. The motor evoked potential amplitude of biceps femoris during dorsi-flexion was significantly increased, whereas the posterior root-muscle reflex amplitude of biceps femoris during dorsi-flexion was significantly decreased compared to rest. These results suggest that corticospinal and spinal reflex excitabilities of soleus are facilitated during knee extension, whereas intra-limb modulation of biceps femoris during dorsi-flexion appeared to be inverse between corticospinal and spinal reflex circuits.

## Introduction

The spinal motoneurons receive excitatory and inhibitory synaptic inputs from sensory afferents and inputs from supraspinal centers either directly or via interneurons. To assess the neural modulation of spinal motoneurons, the excitability of corticospinal and monosynaptic spinal reflex pathways has been addressed using transcranial magnetic stimulation (TMS) and peripheral nerve stimulation, respectively (McNeil et al. 2013). Owing to differences in the gain of these pathways, motor evoked potentials (MEPs) by TMS and the Hoffman-reflex (H-reflex) are modulated differently among the muscles during voluntary contraction (Morita et al. 2000). More specifically, MEPs and the H-reflex of soleus (SOL) were facilitated with increases in plantar-flexor torque, and MEPs of tibialis anterior (TA) were also facilitated, but the H-reflex of TA was not facilitated with increases in dorsi-flexor torque (Morita et al. 2000).

It has been reported that voluntary muscle contraction (e.g., quadriceps femoris) modulates the MEPs and the H-reflex of the other muscles located at other segments within a limb (e.g., triceps surae) (the present study called “intra-limb modulation” below), not only those of contracted muscles (Hwang et al. 2000; Izumi et al. 2001). Previous studies showed that intra-limb modulation of the H-reflex differs between thigh and lower-leg muscles during isometric voluntary contractions (Hwang et al. 2000; Izumi et al. 2001). More specifically, H-reflex depression is observed in SOL during knee flexion (i.e., hamstring muscles contraction) (Izumi et al. 2001), whereas H-reflex facilitation is observed in vastus medialis during plantar-flexion (i.e., triceps surae contraction) (Hwang et al. 2000). These studies suggest that intra-limb modulation of the H-reflex occurs inversely between contracted and tested muscles in the lower limb. The present study assumed that different neural mechanisms are involved in these inverse modulation patterns. However, differences in intra-limb modulation of spinal reflex excitabilities among the lower-limb muscles have remained unknown, due to technical difficulty in evoking spinal reflexes from multiple muscles. The present study used transcutaneous spinal cord stimulation (tSCS) to evoke the posterior root-muscle (PRM) reflexes from the lower-limb muscles simultaneously (Minassian et al. 2007). The tSCS mainly activates the posterior root of the spinal cord, and the evoked responses appear to have characteristics similar to the H-reflex (Courtine et al. 2007). By applying tSCS, the present study could clarify the differences in intra-limb modulation of PRM reflexes among the lower-limb muscles, including the muscles in which it is difficult to evoke the responses by the H-reflex (e.g., hamstrings).

Corticospinal excitability during voluntary contraction can be evaluated by examining the magnitude of MEPs from the contracted muscle by TMS on the primary motor cortex. Previous studies demonstrated that MEP was facilitated in not only the contracted muscles but also the other non-contracted muscles that are located in different segments within a limb (Brouwer and Ashby 1992; Izumi et al. 2001). For example, hamstring muscles contraction facilitated the MEPs of SOL and TA (Izumi et al. 2001). They suggest the spread of the facilitatory effects on the lower leg during hamstring muscles contraction. Assuming the density of cortical projections onto the spinal motoneurons differs among the lower-limb muscles, combining tSCS and TMS techniques may be able to show to what extent the corticospinal pathways are involved in the intra-limb modulation of spinal reflex circuits.

The purpose of the present study was to identify differences in the involvement of corticospinal pathways in intra-limb modulation of spinal reflex circuits among lower-limb muscles during voluntary contractions. Based on the findings of previous studies (Hwang et al. 2000; Izumi et al. 2001), we hypothesized that (1) PRM reflexes of the thigh muscles would be potentiated during plantar-flexion and dorsi-flexion, and PRM reflexes of lower-leg muscles would be depressed during knee extension and flexion; and (2) MEPs of the thigh and lower-leg muscles would be potentiated during plantar-flexion and dorsi-flexion, and knee extension and flexion, respectively.

## Methods

### Subjects

Ten men (age 26.3 ± 2.8 years, height 175.1 ± 5.0 cm, and weight 67.0 ± 7.5 kg) participated in the present study. The procedure, purpose, risks, and benefits associated with the present study were explained to the subjects and written, informed consent was obtained from all of them. The ethics review committee on experimental research with human subjects of the Graduate School of Arts and Sciences at The University of Tokyo approved the experimental protocols, which were conducted in accordance with the guidelines of the Declaration of Helsinki.

### Isometric contraction tasks

Experiments were conducted while subjects were in the supine position on the bench of a dynamometer (CON-TREX, CMV AG, Dübendorf, Switzerland). This was because preferential recruitment of the sensory fibers was induced by tSCS in the supine position compared to the prone and standing positions (Danner et al. 2016). In the supine position, the hip joint was extended to the anatomical position, and the right knee joint was flexed to 30°. The right ankle joint was plantar-flexed to 10° to relax the dorsiflexor muscles and fixed to the attachment of the dynamometer with non-elastic straps.

Following several warm-up trials, the subjects performed an isometric maximal voluntary contraction (MVC) for approximately 3 s. Isometric voluntary contraction types were plantar-flexion, dorsi-flexion, knee extension, and knee flexion. Two MVC trials were performed to obtain the maximal torque. The inter-trial interval was set to 1 min. If the peak torques between the two trials were different by more than 10%, an additional trial was performed. The trial with the highest peak torque among two or three MVCs was used for the analysis. Following a 2-min rest interval, voluntary contraction at 10% of the MVC level was performed. During isometric contraction tasks, the subjects tried to exert the joint torques by contracting the agonist muscles only (e.g., major agonist action of triceps surae muscles, plantar-flexion). The order of plantar-flexion, dorsi-flexion, knee extension, and knee flexion at 10% of the MVC level was randomized. The subjects were provided visual feedback of 10-Hz low pass-filtered torque signals and the target torque level via a computer monitor using specific software (LabChart 7, ADInstruments, Melbourne, Australia).

### Surface electromyographic recording

Surface electromyographic (EMG) signals were recorded from SOL, TA, vastus lateralis (VL), and the long head of the biceps femoris (BF) in the right leg. Ag–AgCl electrodes (Vitrode F-150S, Nihon Kohden, Tokyo, Japan) with an inter-electrode distance of 20 mm were used for EMG acquisition from each muscle. The amplifier was set to a gain of 1000-fold with a bandpass filter between 5 Hz and 1 kHz (AB-611 J, Nihon Kohden). The EMG signals and torque signals were simultaneously sampled at 4 kHz using an AD converter (PowerLab, ADInstruments, Melbourne, Australia) and stored on a personal computer using software (LabChart 7, ADInstruments).

### TMS

A double-cone coil (outside diameter of 110 mm) was placed over the leg area of the left motor cortex to obtain MEPs from the right SOL, TA, VL, and BF by TMS (Magstim 200 stimulator, Magstim, Dyfed, UK). At the beginning of the measurements, the optimal stimulating site (i.e., “hot spot”) providing the largest amplitude for the SOL evoked response was identified. The head of each subject was secured on a head rest. The TMS coil position was marked on the non-elastic cap to ensure that the same area of the cortex was stimulated throughout the experiment. Next, the resting motor threshold (RMT) was determined while subjects rested quietly. The RMT was defined as the lowest stimulation intensity for which peak-to-peak amplitudes of MEP were larger than 50 μV for at least three of five stimuli. The TMS intensity was set to 130% of the RMT of SOL (Abdelmoula et al. 2016). The stimulation intensity in the present study was 76.4 ± 14.7% of maximal stimulator output. MEPs were evoked during the resting and weak voluntary contraction conditions, and ten stimuli with an inter-pulse interval of 6 s were delivered in each condition.

### tSCS

**Fig. 1:**
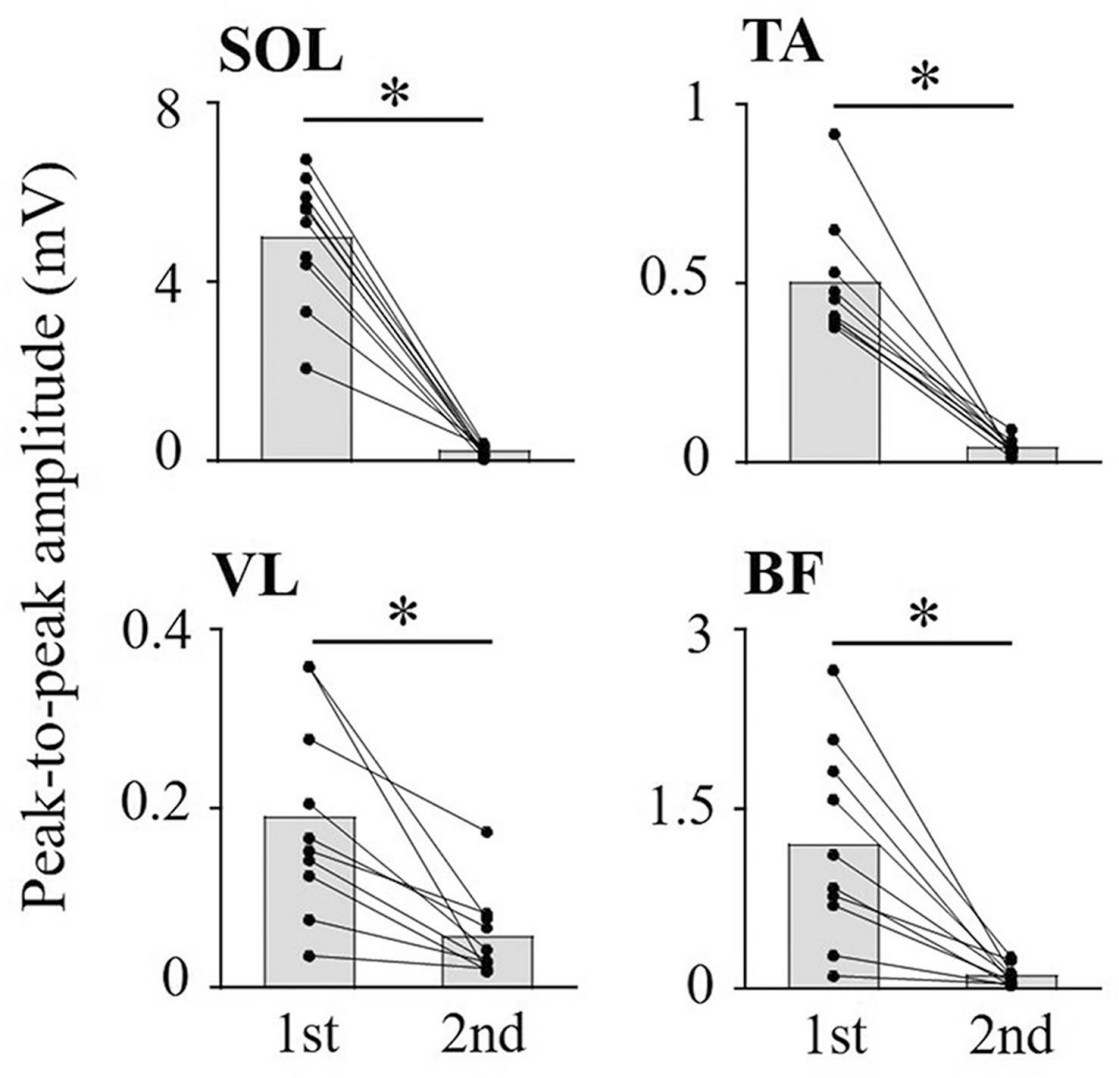
The evoked responses using double-pulse stimulation of tSCS. Gray bars indicate mean values across subjects. Each black circle represents a data point in each subject. *p < 0.05

PRM reflexes in the lower-limb were evoked using a constant current electrical stimulator with a single rectangular pulse of 1-ms duration (DS7A, Digitimer, Hertfordshire, UK). An anode (100 × 75 mm) was placed over the midline of the abdomen between the xiphoid process of the sternum and the umbilicus, and a cathode (50 × 50 mm) was placed on the midline of the back between spinous processes of upper-lumbar vertebrae. Since the stimulation electrode position of tSCS at different spinal levels affects recruitment of the evoked responses (Danner et al. 2011; Roy et al. 2012), in the present study, the cathodes were positioned where larger responses were evoked from all recorded muscles at any stimulation intensity, based on the visual determination of the response magnitude (Masugi et al. 2016; Nakagawa et al. 2018; Saito et al. 2019). The cathode was placed between L1 and L2 in all subjects. To confirm that the responses were initiated in the sensory fiber based on suppression of the second response owing to post-activation depression (Andrews et al. 2015), a double-pulse stimulation with a 50-ms inter-pulse interval and with various stimulation intensities were delivered while the subject was resting (Courtine et al. 2007; Minassian et al. 2007) (Fig. 1). Test stimulation intensity of tSCS was visually determined based on the procedure below. Stimulation intensity was increased from 10 to 20 mA below test stimulation intensity until the appearance of a second response in some muscles by 2-mA stimulation intensity increments. Then, the strongest stimulation intensity that could produce a large first response without a second response for SOL, TA, VL, and BF was chosen. The mean stimulation intensity for tSCS was 61.4 ± 12.2 mA. Under the resting and voluntary contraction conditions, ten stimuli with an inter-pulse interval of 6 s were delivered.

### Data analysis

**Fig. 2:**
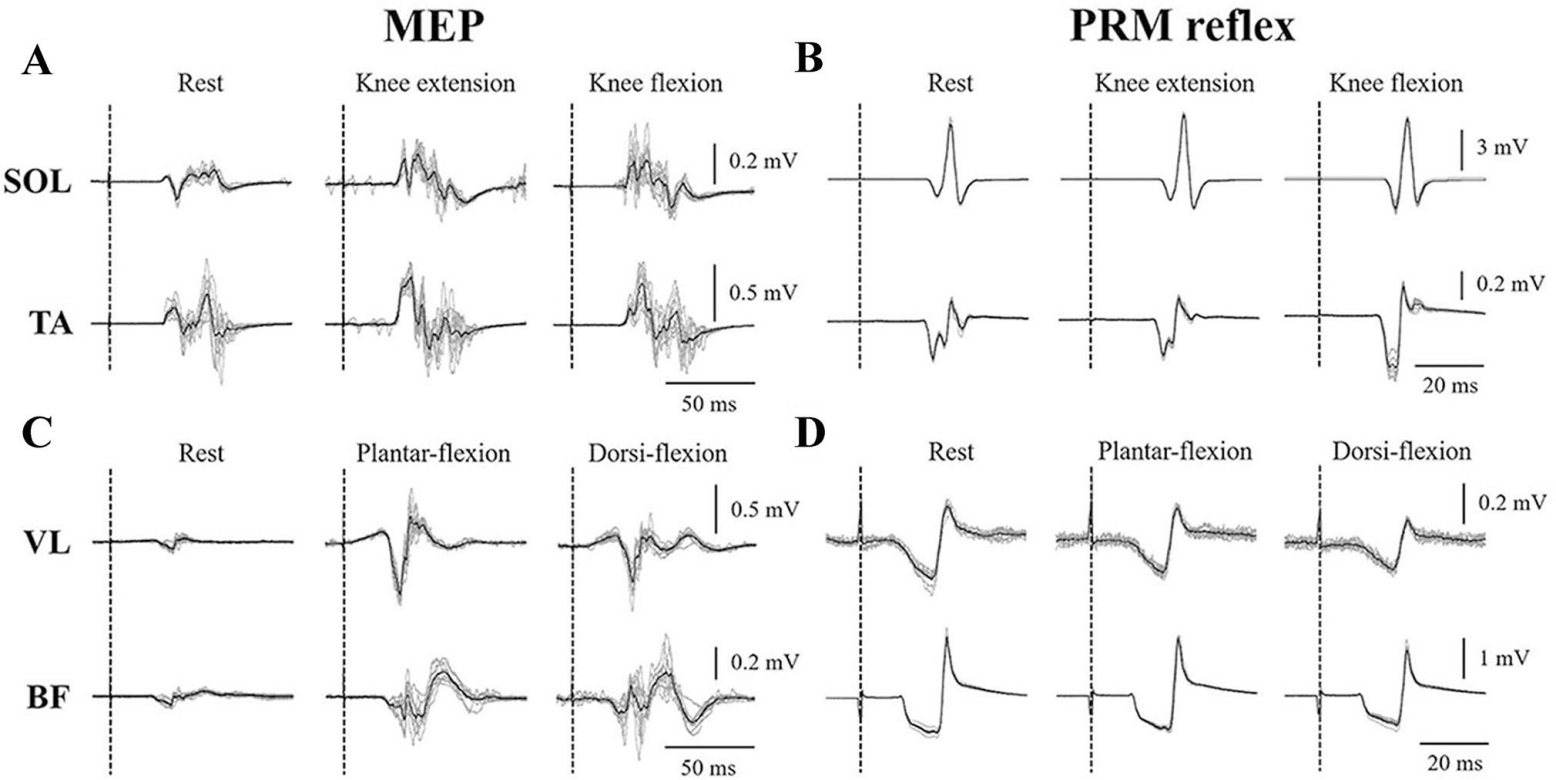
Typical example waveforms of MEPs and PRM reflexes from a single subject. MEPs and PRM reflexes are evoked from SOL and TA during rest, knee extension, and knee flexion (**A** and **B**). MEPs and PRM reflexes are evoked from VL and BF during rest, plantar-flexion, and dorsi-flexion (**C** and **D**). Thin grey lines represent ten MEPs and PRM reflex waveforms overlaid at each trial, and the thick black line is the mean waveform of the MEP and PRM reflex over ten trials. Vertical dotted lines indicate the timing of the test stimulus by TMS and tSCS

The root-mean-square (RMS) values of EMG signals of agonist muscles during MVC were calculated over 1000-ms during the same period that the peak torque was obtained. This was used for normalization of the EMG signals during agonist muscle contraction at the 10% of MVC level. As the background EMG, the RMS in a 500-ms window just before the stimulation by TMS and tSCS was calculated (Duclay et al. 2011; Saito et al. 2021). This was because a window of at least 200-ms is needed for the analysis given the resolution of the high-pass filtered EMG signals in the present study (i.e., cut-off frequency: 5 Hz). Peak-to-peak amplitudes of the MEPs and PRM reflexes during rest, knee extension, and flexion were calculated for SOL and TA, and peak-to-peak amplitudes during rest, plantar-flexion, and dorsi-flexion were calculated for VL and BF (Fig. 2). The amplitudes of MEPs and PRM reflexes across 10 trials were averaged for each condition.

### Statistical analysis

The normality of the data distribution was investigated using the Kolmogorov–Smirnov test, and since the distribution of the data was partly non-Gaussian, non-parametric statistical tests were used. The amplitudes between the first and second responses evoked by tSCS with a double-pulse were compared using the Wilcoxon signed-rank test. Background EMG, MEP, and PRM reflex amplitudes of SOL and TA were analyzed among the three conditions (i.e., rest, knee extension, and flexion) by the Friedman test. Background EMG, MEP, and PRM reflex amplitudes of VL and BF were analyzed among the three conditions (i.e., rest, plantar-flexion, and dorsi-flexion) by the Friedman test. When a significant effect was found by the Friedman test, Scheffé’s test was performed as a *post-hoc* test for the pairs between resting and voluntary contraction conditions (i.e., rest and knee extension or flexion for SOL and TA; rest and plantar-flexion or dorsi-flexion for VL and BF). Data are expressed as means ± SD in the text.

## Results

### Double-pulse stimulation of tSCS

A double-pulse stimulation of tSCS showed that the amplitudes of second responses were significantly lower than those of the first responses for each muscle (SOL: *z* = − 2.8, *p* = 0.005, TA: *z* = − 2.8, *p* = 0.005, VL: *z* = − 2.8, *p* = 0.005, BF: *z* = − 2.8, *p* = 0.005) (Fig. 1). The second response amplitudes relative to the first responses were: SOL 5.1 ± 4.4%; TA 9.0 ± 6.0%; VL 33.7 ± 20.2%; and BF 12.6 ± 11.3%.

### Background EMG activity level during voluntary contractions

**Fig. 3:**
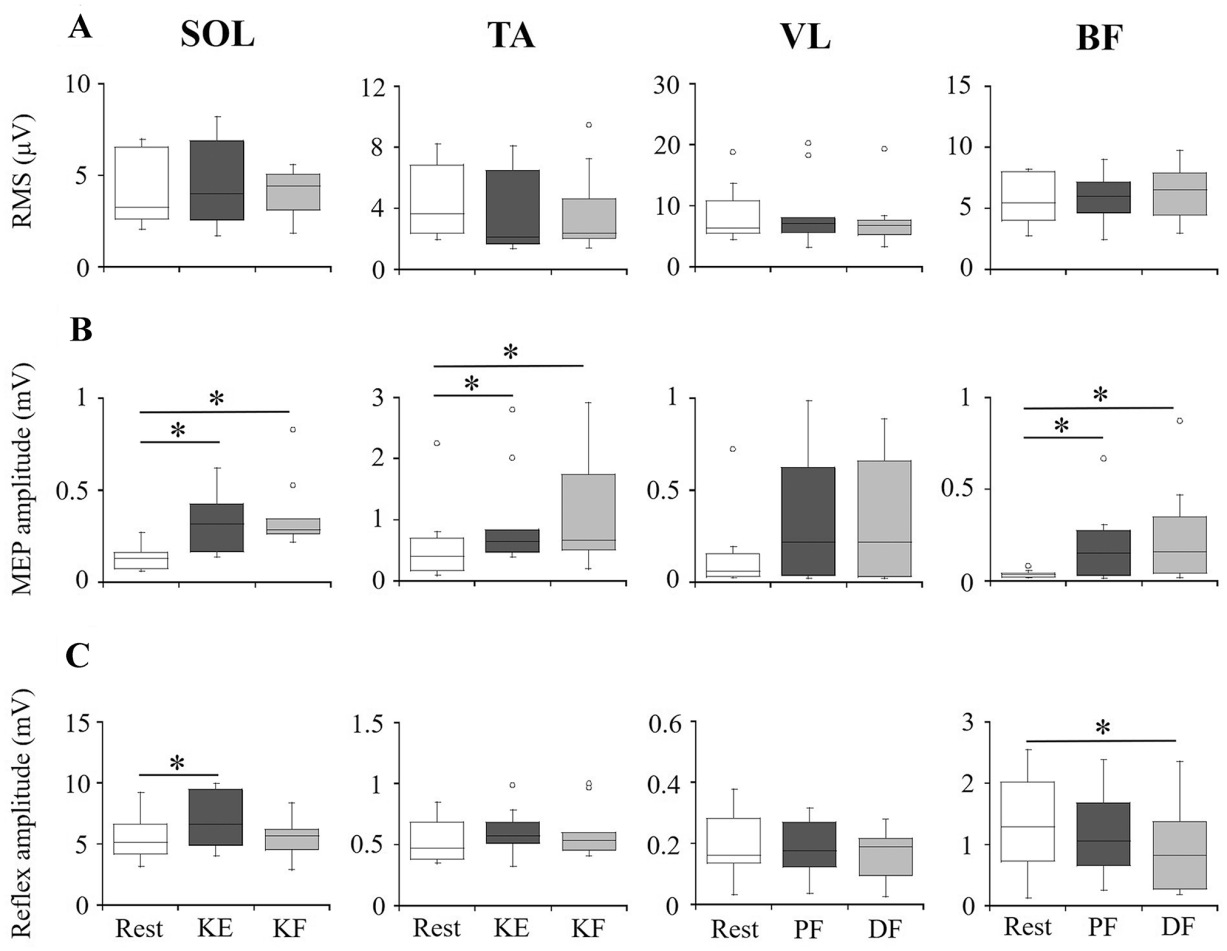
Amplitudes of background EMG, MEP, and PRM reflexes of lower-limb muscles. **A** RMS of background EMG activity; **B** peak-to-peak amplitude of MEP response; **C** peak-to-peak amplitude of PRM reflex response. *KE* knee extension; *KF* knee flexion; *PF* plantar-flexion; *DF* dorsi-flexion. The box plots indicate the median values with upper and lower quartiles. The whiskers represent the higher and lower extreme values, and the outliers are plotted as open circles. *p < 0.05

No significant differences in the background EMG activity among rest, knee extension, and knee flexion were observed in SOL (*χ*^2^_(2)_ = 2.1, *p* = 0.368) and TA (*χ*^2^_(2)_ = 1.5, *p* = 0.459) (Fig. 3A). During knee extension and knee flexion, the EMG activity level of VL and BF as agonist muscles was 18.8 ± 9.7 μV (6.5 ± 2.1% of RMS during MVC) and 21.4 ± 11.3 μV (8.9 ± 3.4% of RMS during MVC), respectively.

No significant differences in the background EMG activity among rest, plantar-flexion, and dorsi-flexion were observed in VL (*χ*^2^_(2)_ = 1.4, *p* = 0.497) and BF (*χ*^2^_(2)_ = 3.2, *p* = 0.197) (Fig. 3A). During plantar-flexion and dorsi-flexion, the EMG activity level of SOL and TA as agonist muscles was 25.5 ± 12.7 μV (15.1 ± 6.6% of RMS during MVC) and 33.3 ± 17.8 μV (8.3 ± 3.8% of RMS during MVC), respectively.

### Intra-limb modulation of MEPs

MEPs of SOL and TA were potentiated during knee extension and flexion, and MEPs of BF were potentiated during plantar-flexion and dorsi-flexion (Figs. 2, 3B). Significant main effects by the Friedman test were observed in MEP amplitudes of SOL (*χ*^2^_(2)_ = 15.2, *p* < 0.001), TA (*χ*^2^_(2)_ = 12.2, *p* = 0.001), and BF (*χ*^2^_(2)_ = 8.6, *p* = 0.014), but not VL (*χ*^2^_(2)_ = 4.200, *p* = 0.078). MEP amplitudes of SOL and TA were significantly higher during knee extension (SOL: *χ*^2^_(1)_ = 10.3, *p* = 0.005, TA: *χ*^2^_(1)_ = 6.5, *p* = 0.038) as well as during flexion (SOL: *χ*^2^_(1)_ = 11.1, *p* = 0.003, TA: *χ*^2^_(1)_ = 8.6, *p* = 0.010) than during rest (Fig. 3B). The MEP amplitude of BF during plantar-flexion (*χ*^2^_(1)_ = 8.6, *p* = 0.010) and dorsi-flexion (*χ*^2^_(1)_ = 14.2, *p* < 0.001) were significantly higher than during rest.

### Intra-limb modulation of PRM reflexes

The PRM reflex of SOL was potentiated during knee extension, whereas the reflex of BF was depressed during dorsi-flexion (Figs. 2, 3C). Significant main effects by the Friedman test were observed in PRM reflex amplitudes of SOL (*χ*^2^_(2)_ = 7.4, *p* = 0.025) and BF (*χ*^2^_(2)_ = 3.6, *p* = 0.020), but not in TA (*χ*^2^_(2)_ = 1.4, *p* = 0.497) and VL (*χ*^2^_(2)_ = 4.2, *p* = 0.122). The PRM reflex amplitude of SOL was significantly higher during knee extension than during rest (*χ*^2^_(1)_ = 7.9, *p* = 0.018), and the PRM reflex amplitude of SOL during knee flexion did not change significantly from rest (*χ*^2^_(1)_ = 0.04, *p* = 0.979). The PRM reflex amplitude of BF was significantly lower during dorsi-flexion than during rest (*χ*^2^_(1)_ = 7.9, *p* = 0.018), and the PRM reflex amplitude of BF during plantar-flexion did not change significantly from rest (*χ*^2^_(1)_ = 0.3, *p* = 0.833).

## Discussion

The purpose of the present study was to identify differences in the involvement of corticospinal pathways in intra-limb modulation of spinal reflex circuits among lower-limb muscles during voluntary contractions. The findings of intra-limb modulation of PRM reflexes were that PRM reflexes of SOL were potentiated during knee extension, and PRM reflexes of BF were depressed during dorsi-flexion. These results did not support our first hypothesis. The findings of intra-limb modulation of MEPs were that MEPs of SOL and TA were potentiated during knee extension and flexion, and MEPs of BF were potentiated during plantar-flexion and dorsi-flexion. These results support our second hypothesis.

### Methodological considerations

It is considered that the evoked responses by tSCS involve the activation of sensory (i.e., posterior root), motor (i.e., anterior root), or mixed sensory-motor fibers (Danner et al. 2016). The evoked responses are affected by the body posture of subjects, stimulation electrode position, and stimulation intensity of tSCS (Roy et al. 2012; Danner et al. 2016). Based on these previous studies, the optimal setup of tSCS was performed to activate the posterior root of the spinal cord. Consequently, the present study confirmed that responses induced by second stimulation of all muscles was strongly depressed by a conditioning stimulus applied 50-ms before the test stimulus (Fig. 1). Therefore, it is considered that evoked responses by tSCS from the lower-limb muscles reflect the excitability of spinal reflex circuits and the modulation of each muscle during voluntary contractions.

Differences in the amplitudes of MEPs and PRM reflexes between lower-limb muscles may affect the modulation of MEP and PRM reflexes evoked by tSCS during voluntary contractions. It should be considered that sensitivity to modulate the responses was different between the lower-limb muscles. This was because the present study addressed the modulation of MEPs and PRM reflexes by one stimulating intensity of TMS and tSCS, respectively. For instance, the sensitivity of the H-reflex to potentiation and depression is related to the magnitude of the test H-reflex (Crone et al. 1990). The present study showed that MEP of TA was larger than of SOL, VL, and BF (Fig. 3B), and the PRM reflex of SOL evoked by tSCS was larger than of TA, VL, and BF during resting and voluntary contraction conditions (Fig. 3C). Hence, the magnitude of MEPs and PRM reflexes would be a potential factor causing differences in intra-limb modulation of corticospinal and spinal reflex circuits among the lower-limb muscles.

One of the potential factors affecting the MEP and PRM reflex amplitudes is the changes of background EMG activity levels of the tested muscles between resting and voluntary contraction conditions. In the present study, the subjects could perform knee extension and knee flexion without muscle activity of SOL and TA, and plantar-flexion and dorsi-flexion at 10% of the MVC level without muscle activity of VL and BF (Fig. 3A). Thus, the effects of background EMG activity of the tested muscles on the intra-limb modulation of MEPs and PRM reflexes during voluntary contractions were negligible.

### Intra-limb modulation of MEPs and PRM reflexes

The present study showed that corticospinal excitability of SOL and TA is facilitated during knee extension and flexion (Fig. 3B), and excitability of the spinal reflex circuit of SOL is facilitated during knee extension (Fig. 3C). A previous study demonstrated that isometric knee flexion facilitates MEPs of SOL and depresses the H-reflex of SOL (Izumi et al. 2001). However, in the previous study, increased background EMG activity was observed in SOL and TA during knee flexion (Izumi et al. 2001). In that case, the background EMG level affected the spinal reflex excitability of SOL, and it also induces another possibility that increased TA muscle activity can inhibit spinal reflex excitability of SOL (i.e., reciprocal inhibition) (Crone et al. 1987). Since muscle activity was not obtained from SOL and TA during knee extension and flexion (Fig. 3A), facilitation of the direct corticospinal pathway to the motoneurons is one of the potential mechanisms inducing intra-limb modulation of the PRM reflex of SOL. Regarding the differences in intra-limb modulation of PRM reflexes between SOL and TA (Fig. 3B), the PRM reflex of TA by tSCS may have been less sensitive to modulation during voluntary contractions (Hofstoetter et al. 2008; Saito et al. 2021).

Neural factors of the “remote effect”, a phenomenon of muscle contraction that can modulate corticospinal excitability of the contracted muscles and other muscles located in a remote segment, may have been involved (Tazoe et al. 2005; Sasaki et al. 2018). For example, facilitatory effects of remote muscle contraction (e.g., teeth clenching) on the H-reflex were observed in the SOL (Miyahara et al. 1996; Zehr and Stein 1999) and TA (Takada et al. 2000). It has been suggested that voluntary teeth clenching induces attenuation of presynaptic inhibition of Ia terminals (Zehr and Stein 1999) and reciprocal inhibition of the motoneurons of SOL (Takada et al. 2000). Thus, in the present study, voluntary contraction of the quadriceps femoris may have induced the attenuation of presynaptic inhibition and reciprocal inhibition of SOL. Furthermore, changes of afferent inputs of sensory receptors can modulate the excitability of spinal reflex circuits. It appeared that facilitatory effects of heteronymous Ia connections (i.e., neural inputs from other Ia afferents) were obtained in the pathways from quadriceps femoris to SOL and TA (Meunier et al. 1990, 1993). Assuming that contraction of quadriceps femoris enhances Ia afferent activity, heteronymous Ia connections from quadriceps femoris to SOL may be one of the explanations for facilitating the PRM reflex of SOL during isometric knee extension.

The present results showed that corticospinal excitability of BF was facilitated during plantar-flexion and dorsi-flexion (Fig. 3B), but spinal reflex excitability of BF was depressed during dorsi-flexion (Fig. 3C). These tSCS results were in line with the previous study showing that the PRM reflex of the hamstrings was smaller during dorsi-flexion than plantar-flexion (Hofstoetter et al. 2008). In the present study, depression of PRM reflexes could not be explained by corticospinal pathways, and some inhibitory neural mechanisms at the spinal level would be involved. First, it is possible that a postsynaptic pathway of the contracted muscles affects the spinal reflex circuits of the other muscles. Antidromic inputs of motoneurons activate Renshaw cell interneurons, causing changes in heteronymous spinal recurrent inhibition. Heteronymous recurrent inhibition was observed from SOL to quadriceps femoris (Iles and Pardoe 1999). In fact, it has been demonstrated that the H-reflex of VL was depressed by conditioning stimulation of the posterior tibial nerve (Iles and Pardoe 1999). Second, various types of sensory receptors distributed in muscles and joints might mediate spinal reflex circuits during isometric contraction of lower-leg muscles. Previous studies demonstrated that heteronymous Ia connection was facilitated in the pathways from SOL to quadriceps femoris and hamstrings, but not in the pathways from TA to quadriceps femoris and hamstrings (Meunier et al. 1990, 1993). Furthermore, the other afferents from sensory receptors (e.g., Ib afferents from Golgi tendon organs) project not only the origin but also the other muscles. In the cat hind limb, it has been reported that inter-segmental inhibitory force feedback linkages are distributed between the thigh and lower-leg muscles (Wilmink and Nichols 2003). Therefore, the depression of the PRM reflex of BF during dorsi-flexion might be caused by afferent inputs to spinal reflex circuits via interneurons. If so, it is possible that depression of the PRM reflex of VL is less than that of BF because the inhibitory effects through spinal interneurons on the motoneurons are weaker in quadriceps than in hamstring muscles (Bayoumi and Ashby 1989). Third, depression of the PRM reflex of BF is likely to be mediated by presynaptic mechanisms. Although the H-reflex modulation of SOL following the quadriceps femoris contraction originated from the heteronymous Ia afferents to SOL motoneurons (Hultborn et al. 1987), the extent of presynaptic inhibition of spinal motoneurons of VL and BF when lower-leg muscles contract is unclear.

From the functional viewpoint, differences in intra-limb modulation of spinal reflex excitabilities among the lower-limb muscles may result from their functional peculiarities in human movements (e.g., antigravity muscles). In the present study, enhancement of the PRM reflex was observed in an antigravity muscle (i.e., SOL), and suppression of the PRM reflex was observed in BF, but not in an antigravity muscle (i.e., VL) (Fig. 3C). It has been reported that reciprocal inhibition is weaker from hamstrings to quadriceps femoris than the reverse (Bayoumi and Ashby 1989). Namely, the antigravity muscles (i.e., triceps surae and quadriceps femoris) could be less sensitive to inhibit the motoneurons. In addition, a previous study demonstrated that recurrent inhibition from quadriceps femoris to TA is similar between walking and standing, but recurrent inhibition of SOL was attenuated during the early stance phase of walking (Lamy et al. 2008). Such modulation may assist the activation of SOL during the stance phase of walking, for its deactivation. Therefore, differences in intra-limb modulation of spinal reflex circuits between SOL and TA and between VL and BF may be attributed to their anatomical and functional roles of individual muscle groups during voluntary movements.

In conclusion, the present study showed that MEPs of SOL and BF were potentiated, and the PRM reflex of SOL was potentiated during knee extension, whereas the PRM reflex of BF was depressed during dorsi-flexion. These results suggest that corticospinal and spinal reflex excitabilities of SOL are facilitated during knee extension, whereas intra-limb modulation of BF during dorsi-flexion appeared to be inverse between the corticospinal and spinal reflex circuits. Elicitation of the responses from multiple muscles by combining TMS and tSCS indicated that the involvement of corticospinal pathways in intra-limb modulation of spinal reflex circuits differed among lower-limb muscles. Differences in intra-limb modulation among the lower-limb muscles during voluntary contractions might be attributed to both descending and peripheral inputs of individual muscles.
